# Subtype AD Recombinant HIV-1 Transmitted/Founder Viruses Are Less Sensitive to Type I Interferons than Subtype D

**DOI:** 10.3390/v17040486

**Published:** 2025-03-28

**Authors:** Denis Omara, Fortunate Natwijuka, Anne Kapaata, Frank Kato, Laban Kato, Christian Ndekezi, Angella Nakyanzi, Mercy L. Ayebale, Ling Yue, Eric Hunter, Obondo J. Sande, Christina Ochsenbauer, Pontiano Kaleebu, Sheila N. Balinda

**Affiliations:** 1 Department of Immunology and Molecular Biology, School of Biomedical Sciences, College of Health Sciences, Makerere University, Kampala P.O. Box 7062, Uganda; omaradenis2@gmail.com (D.O.); fortunate.natwijuka@mrcuganda.org (F.N.); frank.kato@mrcuganda.org (F.K.); christian.ndekezi@mrcuganda.org (C.N.); ojsande@gmail.com (O.J.S.); 2 Medical Research Council, Uganda Virus Research Institute & London School of Hygiene and Tropical Medicine (MRC/UVRI & LSHTM), Uganda Research Unit, Entebbe P.O. Box 49, Uganda; anne.kapaata@mrcuganda.org (A.K.); laban.kato@mrcuganda.org (L.K.); mercylorna20@gmail.com (M.L.A.); pontiano.kaleebu@mrcuganda.org (P.K.); 3 Uganda Virus Research Institute (UVRI), Entebbe P.O. Box 49, Uganda; 4 Emory Vaccine Center, Emory National Primate Research Center, Atlanta, GA 30329, USA; lyue2@emory.edu (L.Y.); ehunte4@emory.edu (E.H.); 5 Department of Pathology and Laboratory Medicine, Emory University, Atlanta, GA 30322, USA; 6 Department of Medicine, University of Alabama at Birmingham, Birmingham, AL 35294, USA; christinaochsenbauer@uabmc.edu

**Keywords:** HIV-1, transmitter founder viruses, type one interferons, resistance, sensitivity

## Abstract

Initial interactions between HIV-1 and the immune system at mucosal exposure sites play a critical role in determining whether the virus is eliminated or progresses to establish systemic infection. The virus that successfully crosses the mucosal barrier to establish infection in the new host is referred to as the transmitted/founder (TF) virus. Following mucosal HIV-1 transmission, type 1 interferons (IFN-I) are rapidly induced at sites of initial virus replication. The resistance of TF variants to these antiviral effects of the IFN-I has been studied among HIV-1 subtypes B and C. However, their role in restricting HIV-1 replication among subtypes D and AD recombinant remains unexplored. This study assessed the sensitivity of HIV-1 subtype D and AD recombinant TF viruses to IFN-I by infecting peripheral blood mononuclear cells in vitro with infectious molecular clones of these viruses. Cells were exposed to varying concentrations of interferon-α and interferon-β, and viral replicative capacity was measured using HIV-1 p24 antigen ELISA from culture supernatants. Sensitivity to IFN-I was quantified based on viral replication levels. The results showed that interferon-α was more effective in inhibiting viral replication than interferon-β, regardless of the varying amounts of IFN-I used. However, recombinant AD viruses were found to be more resistant to the antiviral effects of IFN-I compared to subtype D viruses. These findings highlight the differential sensitivity of HIV-1 subtypes AD recombinant and D TF viruses to IFN-I and underscore the potential of IFN-I as a therapeutic strategy to target TF viruses and reduce HIV-1 transmission, particularly in populations where subtype D is prevalent.

## 1. Introduction

Innate immune responses, particularly type I interferons (IFN-I) are the first line of defense against many pathogens, including HIV-1 infection. The IFN-I that are produced in response to an HIV-1 infection include interferon alpha (IFN-α) and interferon beta (IFN-β) [1].

Type I interferons (IFN-I) initiate signaling pathways leading to interferon-stimulated gene (ISG) expression hence the production of Innate Restriction factors (IRFs) [2]. Although the types of IRFs appear numerous, the most well-studied to date include Tripartite Motif Containing 5-α (TRIM5α) (interferes with HIV-1 uncoating) [3], SAM domain and HD domain-containing protein 1 (SAMHD1) and apolipoprotein B mRNA editing enzyme catalytic-like 3 (APOBEC3) (targeting HIV-1 reverse transcription) [2], MX dynamin-like GTPase 2 (MX2) (targeting nuclear entry) [2], schlafen 11 (SLFN11) (targeting transcription) [4], guanylate-binding protein 5 (GBP5) (targeting post-translational modification) [5], and tetherin (targeting HIV-1 release) [6]. These IRFs prevent HIV-1 acquisition at the mucosal level (i.e., vagina and rectum), right through to limiting virus replication once the infection has occurred.

During HIV-1 transmission, a severe bottleneck exists, resulting in infection established by a single or just a few variants known as the transmitted founder (TF) viruses [7]. This may be due to physical and immunological barriers including the type I interferons.

Understanding the molecular basis underlying the successful transmission could improve our vaccine or prophylaxis strategies, which has led to extensive research on the phenotypic properties of the TF HIV-1 variants [7]. This study aims to determine the sensitivity of HIV-1 subtypes AD recombinant and D TF viruses to IFNs-I (IFN-α and IFN-β) antiviral effects in Uganda, where these subtypes predominate.

We hypothesized that AD recombinant HIV-1 TF variants could be more resistant to the antiviral effects of IFN-α and β compared to the subtype D viruses, hence conferring a higher transmission capacity. Addressing/closing this knowledge gap on cell-mediated innate immune responses to HIV-1 infection could inform the rational design of HIV-1 interventions like therapies and vaccines, and it could also enhance our understanding of viral transmission [8,9].

## 2. Materials and Methods

### 2.1. Study Subjects

Eight archived HIV-1 TF virus samples from heterosexual transmission were used in this study. These were samples from the International AIDS Vaccine Initiative (IAVI) Protocol C early-infection cohort study at the Uganda Virus Research Institute (UVRI) [10]. The samples were collected between 2006 and 2011 in Uganda. The inclusion criteria for the samples was an acute/early phase of HIV-1 infection (i.e., plasma samples taken near the time of infection, with a mean estimated time from infection (EDI) of 42 days (range: 11 to 73) [11].

### 2.2. Ethical Consideration

Ethics approvals were sought for the IAVI protocol C from (Uganda Virus Research Institute—Research and Ethics Committee (UVRI-REC) (Ref: GC-127) and additionally, this study was approved by the School of Biomedical Sciences—Research and Ethics Committee (SBS-REC) (Ref: SBS-790) at Makerere University, Uganda. The permission to use the archived blood plasma samples was obtained from the Medical Research Council/Uganda Virus Research Institute and London School of Hygiene and Tropical Medicine (MRC/UVIR and LSHTM), Uganda Research Unit.

### 2.3. Assessment of Mosaic Full-Length Structure of the TF Viruses

We retrieved the full-length genome sequences of eight TF viruses (Table 1), which we previously published in Balinda et al. (2022) [11] and deposited in Genbank. HIV-1 subtype recombination identification was performed using the Recombination Identification Program (RIP) (http://www.hiv.lanl.gov/content/sequence/RIP/RIP.html, accessed on 13 March 2025). The recombinant HIV-1 drawing tool from Los Alamos National Laboratories (https://www.hiv.lanl.gov/content/sequence/DRAW_CRF/recom_mapper.html, accessed on 13 March 2025) was used to generate the recombinant breakpoint maps. The genomes were mapped with key HIV-1 genes (gag, pol, vif, vpr, vpu, tat, rev, env, and nef) using the HXB2 as a reference sequence.

### 2.4. Generation of Virus Stocks

We previously described the derivation of the proviral plasmids encoding the eight TF HIV-1 infectious molecular clones (IMC) used here in Balinda et al. (2022) [11]. Viral stocks of infectious molecular clones (IMC) were generated by transfecting 1.5 µg proviral plasmid DNA into 293T cells (Cat. CRL-11268, ATCC, Manassas, VA, USA) using the Fugene HD transfection reagent (Roche, Madison, WI, USA). Viral supernatants were harvested from 293T cells at 48 h post-transfection by transferring 2 mL of supernatant to 15 conical tubes. The tube was spun at 2000 rpm for 30 min to pellet any cells in the supernatants. Then, the supernatant was aliquoted to cryotubes at 200 µL per tube and frozen at −80 °C [12].

The titer of each viral stock was determined by infecting TZM-bl cells (The TZM-bl cells line was obtained through the NIH HIV Reagent Program, Division of AIDS, NIAID, NIH: ARP-8129 contributed by Dr. John C. Kappes, Dr. Xiaoyun Wu and Tranzyme Inc., Durham, NC, USA) [13] with 5-fold serial dilutions of the virus in the manner previously described [12]. To score for infection, a permanent marker was used to divide the wells of the 24-well plate into quadrants. All blue cells within a field of view were counted, using a total magnification of 200×, once in each of the four quadrants of a well. Infectious units per μL were calculated as follows: [(total# blue cells/4) × 67]/(μL virus added) = IU/μL and the replica wells were averaged. The infectious unit values were used to calculate the amount of virus to be used in PBMC infection in the downstream experiments.

### 2.5. Virus Replication in PBMCs and Type I Interferon Sensitivity

Transmitted/founder (TF) variants that resemble consensus sequences may be preferentially selected during transmission, potentially due to their enhanced in vivo fitness, as suggested by Carlson (2014) et al. [14]. To investigate whether this advantage extends to in vitro replication, we assessed the replicative capacity (RC) of these viruses in peripheral blood mononuclear cells (PBMCs). Since the proportion of infectious to total viral particles varied across stocks, we standardized the input by using a multiplicity of infection (MOI) of 0.05 to ensure comparable initial infectivity rather than equalizing particle numbers. Viral replication dynamics were evaluated by quantifying HIV-1 p24 antigen levels in culture supernatants every 48 h over a 10-day period, following an infection with each virus under different concentrations of recombinant human interferon alpha 2a (IFN-α) (Cat. SRP4594, Sigma-Aldrich, Darmstadt, Germany) and recombinant human interferon beta 1a (IFN-β) (Cat. IF014, Sigma-Aldrich, Darmstadt, Germany); mathematical derivation of RC is described below (2.6).

Peripheral blood mononuclear cells (PBMC) from ten healthy HIV-1 negative blood donors were stimulated with 20 U/mL of recombinant human interleukin-2 (IL-2) (Cat. CTP0023, Invitrogen Inc., Camarillo, CA, USA) and 3 μg/mL of phytohemagglutinin (PHA) (Cat. 11 082 132 001, Roche, Basel, Switzerland) in R10 (Roswell Park Memorial Institute (RPMI) 1640 Medium (Cat. 61870010, Gibco, Grand Island, NY, USA) supplemented with 10% defined Gibco™ Fetal Bovine Serum (FBS), qualified, (Cat. 11573397, São Paulo, Brazil), 1 U/mL penicillin, 1 μg/mL streptomycin (Cat. 15140122, Gibco, São Paulo, Brazil) and 300 μg/mL L-glutamine (Cat. AAJ6057314, ThermoScientific, Waltham, MA, USA) for 72 h at 37 °C. After 48 h, (100 pg/mL, 10 pg/mL, 1 pg/mL, 0.1 pg/mL, 0.01 pg/mL, 0.001 pg/mL, 0.0001 pg/mL and 0 pg/mL) of Recombinant Human Interferon-α2a (Sigma Aldrich, Product # SRP4594, St. Louis, MI, USA) and Recombinant Human Interferon-β Protein (Sigma Aldrich, Product # IF014, St. Louis, MI, USA.) were added to separate portions of cells in a 24-well-plate 24 h prior to infection. To initiate infection, 5 × 10^5^ cells were then infected in 15 mL conical tubes by 2 h spinoculation at 2200 rpm with a MOI of 0.05 based on the TZM-bl titer in duplicate. Cells were then washed twice in 13 mL RPMI, re-suspended in 1 mL of R10 media containing 20 U/mL of IL-2 and plated in a 48-well plate in duplicate each well taking 500 μL.

### 2.6. Determination of HIV-1 Replicative Capacity

Viral replicative capacity (RC) was determined by sampling 50 μL of supernatant every 48 h, starting with a day zero time point, taken 2–3 h after plating virus-inoculated cells to obtain a baseline p24 level for each infection well. The volume removed was replaced with 50 μL of R10 plus 20 U/mL of IL-2. The Alliance HIV-1 p24 antigen ELISA Kit (PerkinElmer, Boston, MA Product # NEK050B001KT) was used to determine the concentration of de novo produced p24 over time. The protocol was modified by loading 10 μL of the supernatant diluted in 190 μL of R10 plus 20 U/mL of IL-2 instead of 200 μL of the supernatant per well as per the kit protocol; obtained Optical Densities (ODs) were adjusted by multiplying them with a dilution factor of 20. The ELISA plates were read off at a wavelength of 490 nm with a reference filter at the wavelength of >630 nm within 15 min after stopping the reaction. The optical densities were translated into p24 antigen concentration using the standard curves that were run along the samples. Then, the concentrations obtained were plotted against time over the 10-day sampling period. From these plots, the areas under the plotted curves were determined. For each virus, the relative RC in the presence of type I interferon versus that of the untreated control was mathematically derived as the ratio of AUC (treated)/AUC (untreated) [14].

### 2.7. Determination of Relative Particle Infectivity of TF Viruses

We defined the relative particle infectivity as the ratio of infectious units, as measured by the virus titer on TZM-bl cells, a standard reporter cell line whose permissivity correlates with that of PBMC [13], over the total amount of virions, measured by determining the p24 antigen concentration of the virus stock using the Alliance HIV-1 p24 Antigen ELISA kit (Cat. NEK050A001KT, PerkinElmer, Boston, MA, USA). Two of the eight samples had low IU/mL, insufficient to determine their relative particle infectivity; therefore, we used the six samples available to determine the relative particle infectivity.

### 2.8. Statistical Analysis

The p24 values were determined using the ELISA reader and imported into the Excel application, from which the viral replicative capacity curves were plotted for each HIV variant tested at different concentrations of interferons α and β. Replication capacity (RC) scores for each variant were calculated by determining the area under the curve. The sensitivity of the HIV-1 TF variants to the antiviral effects of IFN-α and β were determined by calculating the ratio of the RC score in the presence of IFN-I, at a given concentration, to the RC score in the absence of IFN. The statistical significance of the differences in sensitivity to IFN-I between subtype AD recombinant and subtype D was determined using the two-tailed Wilcoxon matched-pairs signed rank sum test. Spearman’s correlation coefficients were used to show the relationship between the concentration of IFN-I and the resistance of TF viruses. Box-and-whisker plots were used to show the median differences in the sensitivity of the TF viruses to the antiviral effect of IFN-α and IFN-β, as well as the different subtypes of the viruses (AD recombinant and D) to the antiviral effect of IFN-I. The Bivariate Granger Causality test was used to determine whether the difference in the interferon concentration was the causation of the observed RC scores.

## 3. Results

Infectious Molecular Clones (IMC), previously derived by Balinda et al. in 2022 [11] from a total of eight HIV-1-positive archived plasma samples from acute/early infections, were used in this study (Table 1). These represent a section of the young Ugandan heterosexual population with a mean age of 31 (ranging from 21 to 58 years), whose major risk factor for HIV infection was due to co-habitation with a seropositive partner who became ultimately infected [11]. The TF viruses were also previously subtyped by Balinda et al. 2022 [11] and the sequences were published in GenBank accession numbers MW006052 to MW006081 (Table 1).

### 3.1. Mosaic Full-Length Structure of the TF Viruses Tested

**Figure 1:**
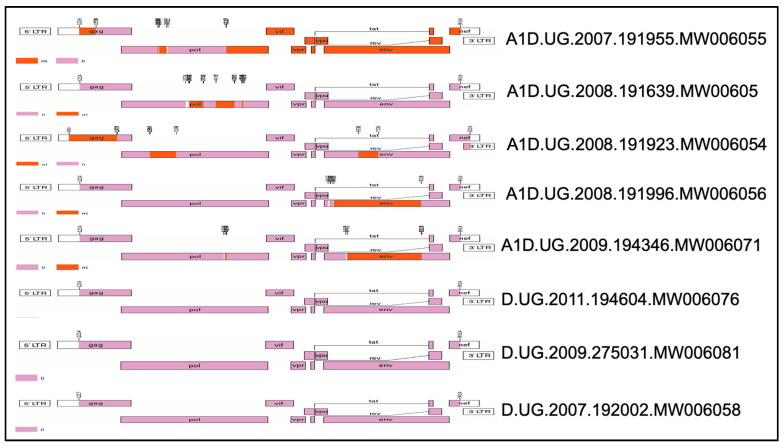
The mosaic full-length structure of the 8 TF HIV-1 tested: Clade A (Purple), Calde D (Orange).

The annotated near-full-length genomes of HIV-1 from Uganda highlight the recombination breakpoints. The genomes were mapped with key HIV-1 genes (gag, pol, vif, vpr, vpu, tat, rev, env, and nef) using the HXB2 as a reference sequence. All A1D recombinants show a mix of subtype A1 (purple) and subtype D (orange). The mosaic full-length structure of the tested TF viruses shows that in four of the five recombinant AD strains most of the genome segments are contributed by subtype D, except A1D.UG.2007.191955.MW006055, which has most of its genome segments coming from subtype A1, with only portions of *gag* and *pol* identified as subtype D (Figure 1).

Recombination breakpoint events occurred within **gag, pol, and env**, with some genomes having additional breakpoints in accessory genes (vif, vpu, tat, rev) (Figure 1). Some genomes have more complex recombination patterns, with multiple breakpoints spread across the genome, as observed in A1D.UG.2008.191923.MW006054 (Figure 1). The pure D subtype genomes do not show recombination, as they appear to be single subtypes (Figure 1). We chose AD recombinants with different recombination breakpoints in an effort to glean information about which region of the genome would affect type I interferon sensitivity.

### 3.2. HIV-1 AD Recombinants Are Less Sensitive to the Antiviral Effects of IFN-I than Subtype D

**Figure 2:**
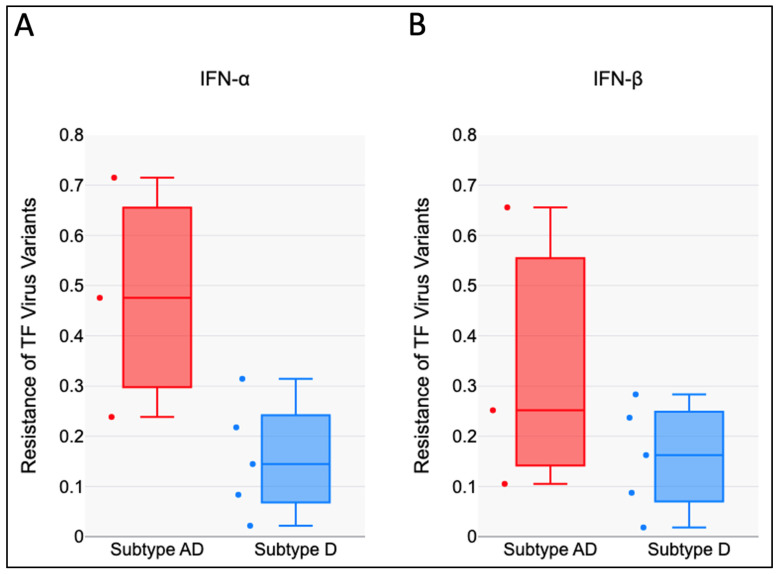
HIV-1 AD recombinants are less sensitive to the antiviral effects of IFN-I than subtype D: difference in the replicative capacity scores between Subtype AD recombinant (red) and Subtype D (blue) in (**A**) IFN-α (t (6) = 2.6, *p* = 0.019) and (**B**) IFN-β (*t* (6) = 1.32, *p* = 0.118).

The RC scores of the five TF subtype AD recombinant variants in the presence of IFN-α were higher than those of the three subtype D (Figure 2A) and the mean difference was statistically significant (*t* (6) = 2.6, *p* = 0.019). The RC Scores of TF subtype AD recombinant variants in the presence of IFN-β also trended higher than those of subtype D (Figure 2B). However, the mean difference was not statistically significant (*t* (6) = 1.32, *p* = 0.118).

### 3.3. Viral Replicative Capacities of TF Viruses Increase with Relative Particle Infectivity

**Figure 3:**
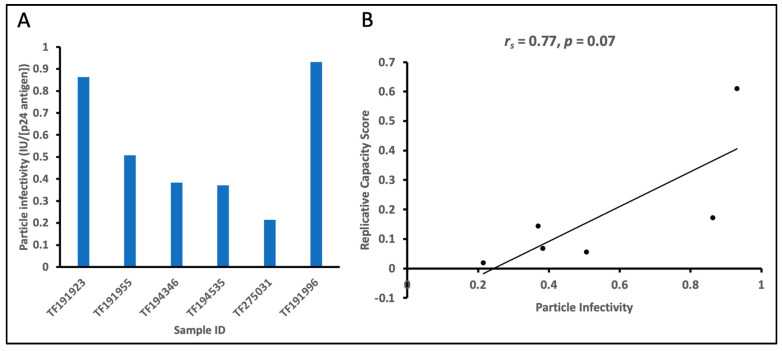
Relative particle infectivity of TF infectious molecular clones. (**A**) The plot for the relative particle infectivity of each infectious molecular clone of the TF viruses. (**B**) Correlation of relative particle infectivity with replicative capacity scores of the TF viruses (r_s_ = 0.77, *p* = 0.07).

In this study, relative particle infectivity is defined as the ratio of virus particles that are able to infect PBMC to the total number of viral particles that are generated from 293T cells. We determined the relative particle infectivity for six of the HIV-1 TF variants; two (TF191639 and TF192002) out of the eight TF IMCs had too low IU/mL for the available volume to derive relative particle infectivity. Analysis of the relative particle infectivity of virus stocks produced from six of the infectious molecular clones showed that they ranged from 0.2 to 0.9 (Figure 3), with four TF viruses having a relative particle infectivity of less than 0.5 (Figure 3A). We confirmed that the RC of virus stocks generated from 293T cells and harvested 48 h after transfection positively correlated with the relative particle infectivity of virus stocks generated from PBMC eight days following infection (Figure 3B; r = 0.77, *p* = 0.07). This shows that the HIV-1 TF variants that are highly infectious on PBMC have higher potential to efficiently replicate.

### 3.4. The TF Viruses Tested Were Less Sensitive to the Antiviral Effect of IFN-β than That of IFN-α

**Figure 4:**
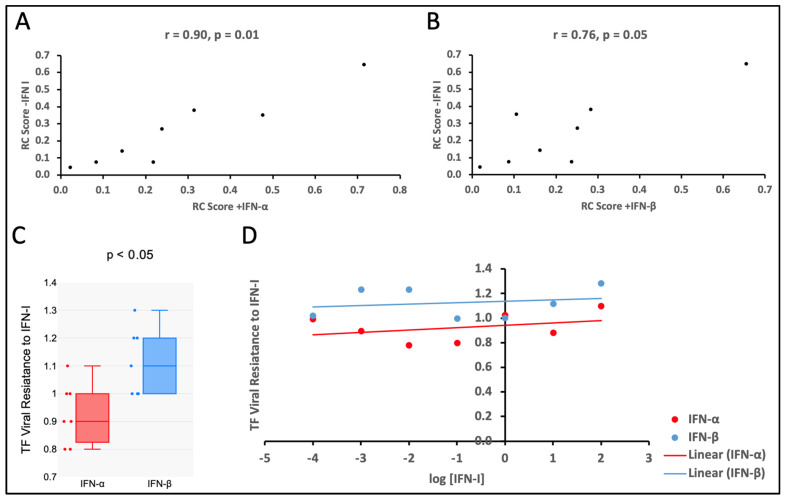
Impact of IFN-α and IFN-β on the replicative capacity of TF viruses’ Spearman’s correlation of the RC Scores in the absence of IFN-I and in the presence of (**A**) IFN-α (r = 0.90, *p* = 0.01) and (**B**) IFN-β (r = 0.76, *p* = 0.05). (**C**) The difference in the resistance of the tested HIV-1 TF variants to the antiviral effects of IFN-α and IFN-β (*p* < 0.05). (**D**) Variation in the resistance of the tested HIV-1 TF variants to the antiviral effects of different concentrations of IFN-α (r = 0.32, *p* = 0.05) and IFN-β (r = 0.21, *p* > 0.05) and causation of the different observed viral replicative capacities by IFN-α (F (7,7) = 3.49, *p* > 0.05) and IFN-β (r = 0.21, *p* > 0.05).

In the eight HIV-1 TF variants tested, the replicative capacity of the viruses in the presence of IFN-α and IFN-β was correlated with their respective in vitro RC scores in the absence of IFN-α and IFN-β (Figure 4A,B; r = 0.90, *p* = 0.01 and r = 0.76, *p* = 0.05, respectively). The statistical significance of the difference between median values of viral sensitivity, as measured by RC, to the antiviral effects of IFN-α and IFN-β was analyzed using a one-tailed Wilcoxon matched-pairs signed rank test. When comparing the medians, the TF virus variants tested were 1.2-fold more resistant to the antiviral effect of IFN-β than to that of IFN-α (Figure 4C; *p* < 0.05). However, the resistance of the viruses was independent of the varying amounts of the IFN-α and IFN-β used to inhibit viral growth, which was consistent with the findings from the previous study by Deymier MJ et al. [8] (Figure 4D; IFN-α F(7,7) = 3.49, *p* > 0.05; IFN-β F(7,7) = 1.24 *p* > 0.05).

## 4. Discussion

The genetic bottleneck is particularly pronounced in heterosexual transmission, where a single viral variant is responsible for most new infections. Understanding the factors influencing transmission may be possible by analyzing the characteristics of these HIV-1 transmitted/founder (TF) variants, which could, in turn, contribute to the development of more effective HIV-1 vaccines and treatments [8]. Research on early and transmitted variants has identified specific genetic and phenotypic features linked to transmission; however, very limited studies have examined the HIV-1 variants prevalent in the East African region, more so the recombinants that are on the increase, specifically in Uganda and Rwanda [15].

When examining the mosaic full-length structure of the TF viruses tested here, we identified breakpoints mainly within *gag*, *pol*, *vpu* and the gp120 coding region of env. Group-specific antigen (*gag*) encodes the structural proteins essential for viral assembly and maturation [16]. The *gag* gene produces a precursor polyprotein (Pr55^Gag) that is cleaved by the viral protease (PR) into four major structural proteins (Matrix, Capsid, Nucleocapsid, and p6) [16]. IFNs induce the expression of innate restriction factors that inhibit HIV replication. Some of these include tetherin MX2, TRIM5α, (BST-2) and APOBEC3 [17]. *Gag* counteracts these effects by optimizing virus assembly and release, ensuring that new virions are efficiently produced despite the IFN-induced restrictions [18]. Recombination at gag breakpoints can shuffle capsid sequences, generating variants with an increased resistance to TRIM5α or MxB and enhancing viral replication in an IFN-rich environment [19].

Besides gag, polymerase (*pol*) is another main region for breakpoints. Several of the breakpoints were within the polymerase (*pol*) region. HIV-1 *pol* encodes three key enzymes: Reverse Transcriptase (RT), which converts viral RNA into DNA; Integrase (IN), which inserts viral DNA into the host genome; and Protease (PR), which cleaves viral gag and *gag-pol* polyproteins for virion maturation [20]. The function of these enzymes may not be directly affected by type one interferons. However, they may disrupt the virus suppression effects of Interferon-Stimulated Genes (ISGs), allowing the virus to persist. In a previous study, it has been hypothesized that the presence of stable and conserved hairpins within the *pol* region may play a role in the higher recombination rates detected at these positions [21].

In addition to gap and pol, Viral Protein U (vpu), an accessory protein encoded by the vpu gene in the HIV-1 genome, is a multifunctional transmembrane protein that plays a key role in enhancing viral replication, immune evasion, and pathogenesis. Vpu downregulates Tetherin, also known as Bone Marrow Stromal Antigen 2 (BST-2) or CD317 [22]. Tetherin is an interferon-induced type II transmembrane glycoprotein [23,24]. Tetherin inhibits the release of enveloped viruses, such as HIV-1, by physically tethering newly budded viral particles to the host cell membrane, hence preventing virus dissemination and promoting viral degradation [24]. If *vpu* genes from certain clades are better in counteracting tetherin than those of others, it could explain why the breakpoints within the vpu region are favored and selected for, as this can result in the *vpu* variants with mutations that enhance its downregulatory effects on tetherin.

The envelope region appears to be a major site for recombination across multiple genomes. The env region of the HIV genome encodes the envelope glycoprotein (gp160), which is cleaved into gp120 (responsible for receptor binding) and gp41 (mediating membrane fusion) [25]. Recombination breakpoints within env can enhance interferon (IFN) resistance by modifying viral entry efficiency, immune evasion, and interaction with host restriction factors [26]. Recombination at the gp120 region of HIV-1 *env* has been associated with reduced sensitivity to type one interferons [11]. Altering the gp120 modifies CD4 and coreceptor (CCR5/CXCR4) binding, allowing the virus to enter cells more efficiently despite the IFN-induced restriction [27]. It also improves Glycan shielding, enhancing protection from IFN-induced neutralizing antibodies [27]. Breakpoints in env can generate recombinant strains with enhanced Viral Infectivity Factor (Vif) function, which degrades APOBEC3G more efficiently, improving viral replication under IFN pressure [28] (APOBEC3G is an antiviral enzyme upregulated by IFN-α that hypermutates viral cDNA, restricting HIV replication [29]).

Away from the breakpoints in the TF HIV genomes, we assessed the viral replicative capacity of three subtype D and five subtype AD recombinants in activated PBMCs, key indicators of in vitro fitness. To explore the possible impact of viral fitness on transmission, we evaluated the in vitro replication efficiency of eight transmitted/founder (TF) variants. The relative particle infectivity of these variants in TZM-bl cells correlated with their replicative capacity in PBMCs, indicating that viral entry into TZM-bl cells reflects an aspect of replication in primary cells [8].

The replicative capacity (RC) varied among the eight transmitted/founder (TF) variants tested. While in vitro RC in activated PBMCs may not accurately represent in vivo transmission fitness, replication in stimulated PBMCs might mimic the inflammatory environment present later in the infection and during the chronic stages, rather than the conditions at the initial sites of viral replication [8]. Transmission of low in vitro fitness variants may seem counter-intuitive; however, the full-length TF IMC, as well as over 200 IMC chimeric for TF Gag have been shown to exhibit a wide range of in vitro RC [30], as found in eight TF viruses in this study. A previous theoretical model of HIV transmission fitness suggested that variants with a lower replicative capacity, that may result in an increased establishment of latency, could have a higher transmission potential in vivo [31]. Therefore, it is possible that a slight reduction in the in vitro replicative capacity could reflect TF virus properties that provide an advantage during transmission.

A potential factor influencing selection during mucosal transmission is the early innate immune response to HIV-1. Innate antiviral cytokines, such as IFN-α, are triggered at the initial sites of HIV-1 replication in the mucosa and draining lymph nodes [32,33]. As a result, the HIV-1 variants that are more resistant to the antiviral effects of IFN-α may have a transmission advantage. For instance, the cross-species transmission of Simian Immunodeficiency Virus (SIV) to humans required evasion of the interferon-stimulated APOBEC3 restriction factors, achieved through enhanced Vif antagonism [34]. A previous comprehensive study using the rhesus macaque model also demonstrated that the IFN-α treatment before intrarectal SIVMAC251 inoculation reduced the number of transmitted variants and increased the number of challenges needed to establish infection [35]. Supporting the idea that type 1 interferons play a role in the transmission bottleneck, prior studies involving HIV-1 showed that transmitted-founder (TF) variants are generally more resistant to IFN-α in vitro than the viruses found during early chronic infection [36,37]. Fenton-May et al. found that TF viruses, from both subtypes B and C, in infected subjects were more resistant to IFN-α compared to variants from the same individual six months post-infection or during early chronic infection [37]. Parrish et al. (2013) also found that TF viruses were more resistant to IFN-α than viruses from the unmatched chronic controls, although this was only true for subtype B and not for subtype C variants [36]. Studies in other laboratories have not observed a consistent difference in IFN sensitivity between transmitted founder viruses and viruses derived from the transmitting partner [38,39,40]. In further studies, a comparison of TF with the non-transmitted HIV variants to determine whether the TF viruses exhibited enhanced resistance to IFN-α and IFN-β compared to NT viruses would give a better conclusion on the transmission fitness of the TF viruses.

We found that the replication of the TF variants in the presence of IFN-α and IFN-β was correlated with in vitro RC scores in the absence of IFN-α and IFN-β, suggesting that in vitro growth in the presence of IFN-α and IFN-β was largely determined by viral replicative capacity. When comparing the median resistance, the TF variants tested were 1.2 folds more resistant to the antiviral effect of IFN-β than that of IFN-α. This finding indicates that IFN-α inhibits TF viral replication in PBMC more effectively than IFN-β. Based on these results, it seems likely that IFN-α contributes more to the HIV-1 transmission bottleneck than IFN-β. However, in a similar study conducted with HIV-1 subtypes B and C by Iyer et al. (2017), IFN-β showed better antiviral potential than IFN-α [41]. This suggests that the antiviral potential of the IFN-I may be subtype-dependent. We also compared the RC Scores of the TF variants of subtype AD recombinants to that of subtype D variants in the presence of IFN-α and IFN-β. Subtype AD recombinant viruses were more resistant to the antiviral effect of both IFN-α and IFN-β than subtype D. However, the resistance of the viruses to IFN-I was independent of the varying amounts of the I IFN-α, and IFN-β used to inhibit viral growth, and this was consistent with the findings from the previous study by Deymier et al. [8]. This means there was either no dose-response or the concentrations of the IFN-I investigated in this study were lower than the minimum response concentration. The impact of the multiplicity of infection on measured interferon resistance has been noted previously [42], thus necessitating the use of a low MOI (less than 0.05).

## 5. Conclusions

In conclusion, our findings reveal differences in the susceptibility of HIV-1 subtypes D and AD recombinant TF viruses to the antiviral effects of IFN-I, with recombinant AD strains showing higher resistance. This underscores the importance of tailoring therapeutic strategies that leverage IFN-I, especially in regions with a high prevalence of these subtypes. The study provides valuable insights into factors that may impact the dynamics of HIV-1 transmission and highlights the potential of IFN-I as a targeted intervention to curb the spread of HIV-1. Future research should further explore the molecular mechanisms underlying these differences to inform the development of more effective treatments and also compare transmitted to non-transmitted viruses from within the quasispecies of chronically infected individuals to better refine and clarify why certain variants became TF variants that successfully establish infection in a new host despite the inhibitory effects of type I interferons (IFN-I). We acknowledge the limitations of our sample size of eight HIV-1 TF viruses only, which may affect the generalizability of our findings. Future studies with larger cohorts are needed to validate and expand upon these results.

## Figures and Tables

**Table 1:** The subtypes and GenBank access numbers of the TF viruses.

Sample ID	Sample Date	Estimated Date of Infection	Days Post Infection	Initial Viral Load (Copies/mL)	Gender	Age	Subtype (Full Genome)	GenBank No
191955	26-Mar-07	03-Mar-07	23	4190	F	39	AD	MW006055
192002	24-Jul-07	04-Jun-07	50	2420	F	27	D	MW006058
275031	05-Jun-09	11-May-09	25	3750	M	31	D	MW006081
191923	24-Jan-08	30-Nov-07	55	198,000	F	31	AD	MW006054
191639	03-Apr-08	13-Feb-08	50	102,000	M	50	AD	MW006052
191996	19-Sep-08	26-Jul-08	55	201	F	37	AD	MW006056
194346	31-Mar-09	28-Feb-09	31	259,000	M	29	AD	MW006071
194604	28-Mar-11	Feb-11	44	254,516	F	35	D	MW006076

## Data Availability

The data presented in this study are available upon request from the corresponding author.
